# Gut Microbiota Modification: Another Piece in the Puzzle of the Benefits of Physical Exercise in Health?

**DOI:** 10.3389/fphys.2016.00051

**Published:** 2016-02-18

**Authors:** Begoña Cerdá, Margarita Pérez, Jennifer D. Pérez-Santiago, Jose F. Tornero-Aguilera, Rocío González-Soltero, Mar Larrosa

**Affiliations:** Research Group on Nutrition, Physical Activity and Health, School of Doctoral Studies and Research, Universidad Europea de MadridMadrid, Spain

**Keywords:** LPS, muscle-microbiota axis, bile acids, myokines, SCFA, IgA, TLR4

## Abstract

Regular physical exercise provides many health benefits, protecting against the development of chronic diseases, and improving quality of life. Some of the mechanisms by which exercise provides these effects are the promotion of an anti-inflammatory state, reinforcement of the neuromuscular function, and activation of the hypothalamic–pituitary–adrenal (HPA) axis. Recently, it has been proposed that physical exercise is able to modify gut microbiota, and thus this could be another factor by which exercise promotes well-being, since gut microbiota appears to be closely related to health and disease. The purpose of this paper is to review the recent findings on gut microbiota modification by exercise, proposing several mechanisms by which physical exercise might cause changes in gut microbiota.

## Introduction

A sedentary lifestyle is associated with a high incidence of chronic diseases such as cardiovascular diseases, type 2 diabetes, cancer, and metabolic syndrome (Owen et al., 2010). Physical exercise is a powerful preventive and treatment tool in several diseases, inducing metabolic and immune effects that provide health benefits. In fact, exercise prescription is effective in preventing ischemic heart disease, stroke, hypertension, colon and breast cancer, type 2 diabetes, metabolic syndrome, osteoporosis, sarcopenia, functional dependence and falls in the elderly, cognitive impairment, anxiety, and depression (Bayego et al., 2012). There are several mechanisms involved in the health-promoting effects of physical exercise, such as the promotion of an anti-inflammatory state, activation of the hypothalamic–pituitary–adrenal (HPA) axis, and reinforcement of neuromuscular function (Gonzalez-Freire et al., 2014; Silverman and Deuster, 2014). When a muscle is contracted with a certain intensity it works as an endocrine organ that releases cytokines (IL-6, IL-8, IL-15) and activates the PPAR coactivator 1 (PGC-1α), which in turn increases mitochondrial biosynthesis and fatty acid utilization (Bishop-Bailey, 2013). Muscle contraction also activates the Forkhead box class O family members FoxO1 and FoxO3 that regulate energy metabolism and govern protein breakdown and muscle mass (Sanchez et al., 2014). In recent years, a new factor by which exercise may promote beneficial health effects has emerged: the modification of gut microbiota. The impact of physical exercise on gut microbiota has barely been revealed. In this review, we outline the potential mechanisms by which exercise could impact gut microbiota.

## Gut microbiota

The gut microbiota is a set of microorganisms that live throughout the gastrointestinal tract of mammals, and which increase in number and diversity from the stomach to the colon. It has been estimated that human microbiota consists of 10^14^ cells (10 times the total number of cells in the human body). In fact, in recent years some authors have defined the human body as a symbiotic super-organism made up of eukaryotic and prokaryotic cells (Eberl, 2010). Microbiota could be composed of 500–1000 different species, the composition of this microbial community is host-specific, evolves throughout the life of the individual, and it may be changed by both exogenous and endogenous stimuli. Gut microbiota composition begins to take shape at birth and continues to do so during lactation. Babies born vaginally have a more diverse microbiota, in which *Lactobacillus, Prevotella*, or *Sneathia* species predominate, while babies born by Cesarean have a less diverse microbiota in terms of genera, with a predominance of *Staphylococcus, Corynebacterium*, and *Propionibacterium* (Dominguez-Bello et al., 2010). In childhood, microbiota adopts the composition of adulthood (Sobhani et al., 2011; Spor et al., 2011) in which only 7–9 phyla of the total phyla that make up the Bacteria domain are represented. In adulthood microbiota, 90% of the phylotypes belong to *Firmicutes* (60–80%) and *Bacteroidetes* (15–30%) phyla (Ley et al., 2006a; Ringel-Kulka et al., 2011), whereas the other minority group of bacteria belongs to *Proteobacteria, Actinobacteria, Fusobacteria*, and *Verrucomicrobia* phyla (Robles Alonso and Guarner, 2013).

## Gut microbiota and health

The relation between gut microbiota and health is increasingly evident. Gut microbiota is crucial for the development of the immune system, in fact, animals raised under absolute sterile conditions (without colonizing microbiota) have a deficient intestinal immune system (Round and Mazmanian, 2009). The bidirectional interaction between microbiota and the host immune system begins at birth and both evolve throughout the life of the host. The intestinal immune system is continuously in contact with gut microbiota and must discriminate between beneficial and harmful bacteria. The immune system through pattern-recognition receptors [like Toll-like receptors (TLRs)] recognizes microbial-associated molecular patterns (MAMPs) expressed in bacteria and depending on the type of bacteria, tolerance is established or an immune response is triggered. Paneth cells and B cells produce anti-microbial peptides and immunoglobulin A respectively, that shape commensal microbiota (Lei et al., 2015). On the other hand, gut microbiota preserves the mucosal barrier integrity by inhibiting the adhesion and growth of enteropathogens, and some types of bacteria have been specifically implicated in mucosal tolerance via induction of immune system T regulatory cells (Thompson-Chagoyán et al., 2007; Brown et al., 2013). The balance between the immune system and commensal microbiota is essential for maintaining health, and the breaking of this equilibrium can trigger many diseases, not only related to the gastrointestinal system, such as ulcerative colitis, Crohn's disease, and colon and gastric cancer (Tamboli et al., 2004; Sobhani et al., 2011; Amirian et al., 2013), but also other diseases such as metabolic syndrome (Delzenne et al., 2011), diabetes type I and type II (Giongo et al., 2011), allergic diseases (atopic eczema/dermatitis and asthma) are associated with a low diversity of gut microbiota and food allergy in infants could be related to a specific microbiota profile (Arrieta and Finlay, 2014; Inoue and Shimojo, 2015), rheumatoid arthritis (Vaahtovuo et al., 2008) and autism (Song et al., 2004; Parracho et al., 2005). The presence of certain bacterial strains of the *Lactobacillus* and *Bifidobacterium* genera in our gut microbiota enhances the absorption of minerals and vitamins, improves lactose intolerance, has anti-diabetic effects, lowers cholesterol levels, increases resistance to pathogen infection, decreases the incidence of colon cancer (Zhu et al., 2011; Kumar et al., 2012), and exerts anti-inflammatory effects at local and systemic levels, improving the development of a controlled and protective immune system (Villena and Kitazawa, 2014). In addition to these functions, the metabolic capacity of gut microbiota is such that it has been called the forgotten organ (O'Hara and Shanahan, 2006), because its metabolism is comparable to that of the liver (Gill et al., 2006). The different communities of bacteria that make up gut microbiota have a variety of metabolic enzymes and other biochemical pathways different from those of the host, which allow them, for example, to synthesize vitamins (K, biotin, folic acid) or ferment indigestible fiber (Legrand et al., 2010; Ou et al., 2012). In recent years, changes in microbiota composition have been associated with obesity (Ley et al., 2006a). Obese individuals have different microbiota composition than their counterparts, *Bacteroidetes* phylum is less represented whereas the proportion *Firmicutes* phylum is increased (Ley et al., 2005, 2006b). Moreover, microbiota transplant from obese mice to lean mice produce obesity in the recipients independently of the food intake (Turnbaugh et al., 2008). A change in microbiota profile toward a population of bacteria that effectively extract more non-assimilable nutrients from the diet, such as plant polysaccharides, could add 10–15% more calories to the energy requirements of the hosts and may therefore influence their obesity (Turnbaugh et al., 2006; Quigley, 2013). Furthermore, microbiota is able to influence hepatic triglyceride production, lipid metabolism (by modulating the pattern of bile acids), carbohydrate metabolism, and systemic low-grade inflammation associated with obesity, insulin resistance, and metabolic syndrome (Backhed et al., 2004; Quigley, 2013). Regarding proteins, some studies indicate that excessive protein fermentation in the colon by harmful bacteria may play a role in colon cancer (Corpet et al., 1995; Toden et al., 2005).

### Aspects influencing gut microbiota

**Figure 1:**
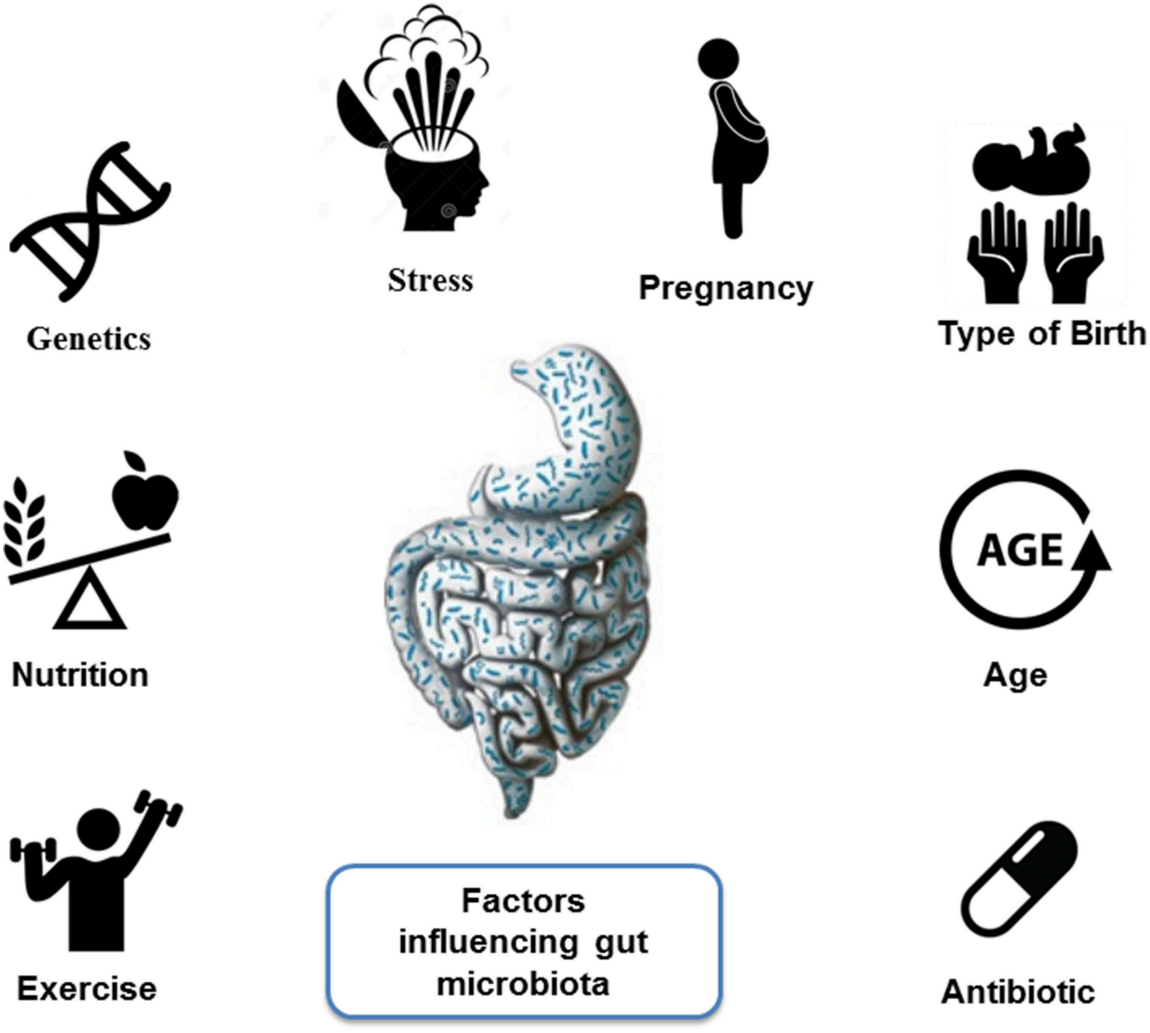
**Factors influencing gut microbiota**.

Gut microbiota is influenced by several factors, including host genetics, age (Dicksved et al., 2008; O'Toole, 2012), pregnancy (Koren et al., 2012), and some environmental factors such as diet (De Filippo et al., 2010; David et al., 2014), the type of birth (Salminen et al., 2004), stress, and antibiotic intake (Nicholson et al., 2012) (Figure 1). Notwithstanding the fact that several factors influence microbiota, it seems that microbiota composition remains relatively constant throughout our lives, although in old age species diversity seems to be lower (Koenig et al., 2011; Yatsunenko et al., 2012). When microbiota is studied at levels below the phylum, a greater variation of microbiota between individuals is observed, and although it is known that microbiota of healthy individuals provides them a number of health benefits, it is not clear what would be the ideal composition of the “healthy microbiota.” It is suspected that the presence of certain species as *Faecalibacterium prausnitzii, Roseburia uniformis*, and *Bacteroides intestinalis* is “key” in shaping a “healthy microbiota” (Qin et al., 2012; Guinane and Cotter, 2013). At present it is not known whether there are other factors that influence microbiota composition, and the scientific community is working hard trying to find which are the predominant factors that modify microbiota; the interrelations between microbiota composition; its pool of bacterial genes (microbiome) and their expressing functions; and the physiological phenotype or disease of the host (Lozupone et al., 2013).

## Physical exercise and gut microbiota

Physical exercise performed at the doses recommended by the World Health Organization (WHO) results in improved fitness, enhancing the quality of life. Exercise is intended as a useful tool to prevent disease and improve the prognosis. Diseases in which exercise promotes a beneficial effect include prostate and ovarian cancer (Cannioto and Moysich, 2015; Wekesa et al., 2015), cardiovascular diseases (Schuler et al., 2013), diabetes (Asano et al., 2014), and stress-related disorders such as anxiety and depression (Silverman and Deuster, 2014). The mechanisms by which exercise has a beneficial effect on health are numerous: effects on the HPA axis, promotion of an anti-inflammatory state, and neuroplasticity augmentation (Silverman and Deuster, 2014). One element that could be positively modified by physical exercise and through which it could promote well-being is the gut microbiota. Although several years ago Bäckhed et al. suggested that there could be a muscle-microbiota axis (Backhed et al., 2007), there are very few studies in the literature that have addressed the modification of gut microbiota by exercise, and all but one have been carried out in murine models. In a recent study, Choi et al. showed changes in the composition of the microbiota in mice which performed exercise vs. sedentary mice. A total of 2510 taxa of bacteria showed differences between the exercise group and the sedentary group. Mice that performed physical exercise showed more abundance of the *Lactobacillales* order, presenting up to 24 times more *Enterococcus faecium* bacteria than sedentary mice, and a marked decrease (-361 fold) of *C11_K211* bacteria of the *Tenericutes* phylum (Choi et al., 2013) (Table 1). These results agree with those of Queipo-Ortuño et al. indicating that exercised rats showed an increase in *Lactobacillus* and *Blautia coccoides–Eubacterium rectale* groups (Queipo-Ortuño et al., 2013). Furthermore, in a study carried out with diverse rat strains, an increase in bacterial diversity in exercised rats was described, and more specifically an increase in *Lactobacillus* genus in obese rats subjected to physical exercise (Petriz et al., 2014). Interestingly, a significantly high inverse correlation between blood lactate concentrations and *Clostridiaceae* and *Bacteroidaeae* families and the *Ruminococcus* genus was found, whereas the *Oscillospira* genus was positively correlated with lactate levels (Petriz et al., 2014). However, the sample size of each experimental group in this study (*n* = 3) compromised the reliability of these results, and thus further studies are needed to confirm them. Surprising data were found when microbiota of mice on a high-fat diet (with and without exercise) and on normal-diet (with and without exercise) were compared (Kang et al., 2014). Exercise not only counteracted the microbiota changes induced by the high-fat diet but caused large shifts in *Firmicutes, Bacteroidetes*, and *Tenericutes* phyla in the same direction and order of magnitude as those caused by the high-fat diet (Kang et al., 2014). Similarly, an additive effect on increasing microbial diversity between a diet high in fat and voluntary exercise was observed in mice (Evans et al., 2014). In this study, exercise increased the percentage of *Bacteroidetes* and decreased *Firmicutes* phyla regardless of diet; moreover, the ratio of *Bacteroidetes*:*Firmicutes* correlated inversely with the amount of performed exercise (Evans et al., 2014). When exercise was applied to healthy and diabetic mice, changes in *Bacteroides*/*Prevotella* spp., *Methanobrevibacter* spp., and *Clostridium cluster I* were observed for both groups, whereas an increase in *Bifidobacterium* spp. level was only observed in exercised non-diabetic mice, indicating that the presence of diabetes nullified this effect (Lambert et al., 2015) (Table 1). These data may indicate that changes induced by exercise are influenced by the metabolic state of the individuals, and this factor must be taken into account in further studies. From a different approach, Hsu et al. observed that mice lacking microbiota, those monocolonized with *Bacteroides fragilis*, and normally raised mice had different exercise performance on a strenuous exercise, and the observed effect seemed to be mediated by the impact of the resident microbiota on the antioxidant status (Hsu et al., 2015). In the unique human study carried out up to now comparing athletes (rugby players) vs. healthy controls, Clarke et al. observed that the athletic group had a greater diversity of microbial species—22 phyla, 68 families, and 113 genera—in contrast with the 11 phyla, 33 families, and 65 genera of the control group (Clarke et al., 2014). However, notwithstanding the differences in the athletes' diets with respect to that of the controls, a unique effect of exercise on gut microbiota diversity could not be determined, taking into account the considerable impact of diet on gut microbiota (Clarke et al., 2014; Flint et al., 2015; O'Sullivan et al., 2015).

**Table 1:** **Physical exercise effects on gut microbiota profiles**.

**Model**	**Exercise**	**Method used to perform metagenomics analysis of gut microbiota**	**Modified bacterial groups**	**References**
Male C57BL/6 mice	Voluntary running wheel 5 w	PhyloChip Array	↓*Tenericutes*	Koenig et al., 2011
			↓*Bacteroidetes*	
			↓*Firmicutes*	
			↑*Lactobacillales*	
Male Sprague Dawley rats	Voluntary running wheel 6 d	V2–V3 regions 16S rRNA PCR-DGGE qPCR	↑Diversity	Yatsunenko et al., 2012
			↑*Actinobacteria*	
			↑*Bifidobacterium*	
			↓*Bacteroides*	
			↓*Prevotella*	
			↓*Firmicutes*	
			↑*B. coccoides- E. rectale group*	
			↓*Enteroccocus*	
			↑*Lactobacillus*	
Obese (Zucker), hypertensive (SHR) and Wistar rats	Treadmill 30 min/d 5 times/w for 4 w	V5-V6 regions 16 rRNA, 454 GS FLX Titanium sequencer platform	↑*Firmicutes*	Guinane and Cotter, 2013
			↓*Proteobacteria*	
			↑*Lactobacillus*	
			↑*Allobaculum*	
			↓*Streptococcus*	
			↓*Sutterella*	
			↓*Aggregatibacter*	
Male C57BL/6 J mice	Running wheel 5 d/w for 24 w	V3–V5 regions 16S rRNA, Miseq Illumina platform	↑*Firmicutes*	Qin et al., 2012
			↓*Tenericutes*	
			↓*Bacteroidetes*	
Male C57BL/6 mice	Running wheel 7d/w for 12 w	V4 region of the 16S rRNA, Miseq Illumina platform qPCR T-RFLP	↑*Bacteroidetes* ↑*Proteobacteria* ↓*Actinobacteria* ↓*Firmicutes*	Lozupone et al., 2013
Male db/db and db/^+^ mice	Low-intensity treadmill running 5d/w during 6 w	qPCR	↑*Bifidobacterium*	Wekesa et al., 2015
			↑*C. leptum CIV*	
			↑*Clostridium cluster I*	
Male rugby players vs. healthy male controls	Observational study	V4 region 16S rRNA 454 Genome Sequencer FLX platform	↑Diversity	Schuler et al., 2013
			↓*Bacteroidetes*	
			↓*Bacteroides*	
			↓*Lactobacillaceae/ Lactobacillus*	
			↑*Akkermansiaceae/Akkermansia*	

*d, days; w, weeks; ↑, increase; ↓, decrease*.

## Potential mechanism by which exercise influences gut microbiota

**Figure 2:**
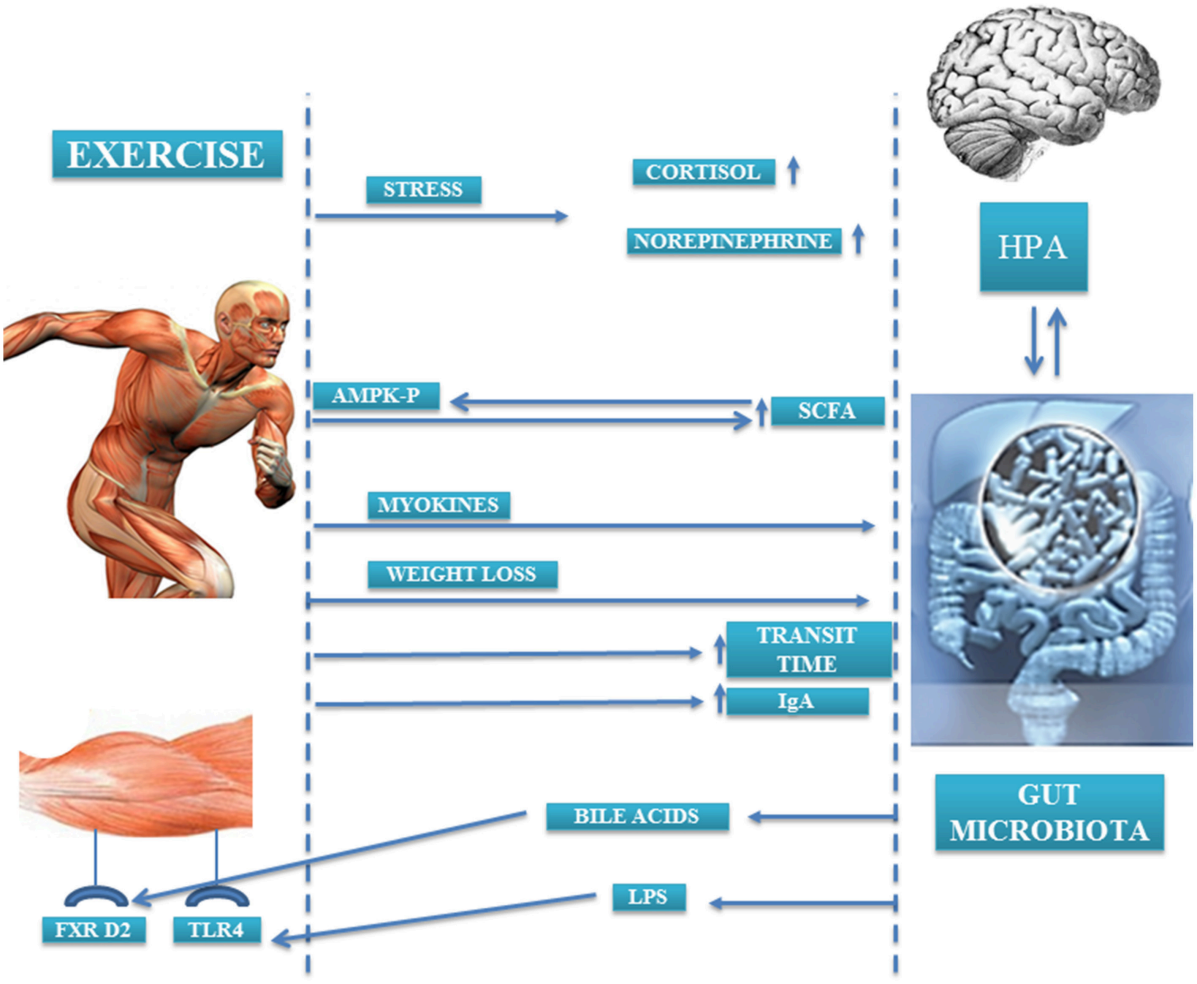
**Gut microbiota-exercise interaction mechanisms**.

In a study conducted by Bäckhed et al. it was observed that animals lacking microbiota were resistant to diet-induced obesity. In the search for mechanisms that could explain this effect, the authors noted that there were two metabolic pathways through which muscle and microbiota were linked. On the one hand, levels of the 5′ adenosine monophosphate-activated protein kinase (p-AMPK), an enzyme involved in energy homeostasis and activation of fatty acid oxidation and glucose uptake in muscle, were 40% higher in the muscle of germ-free rats compared than that of control rats; thus, the presence of microbiota suppressed fatty acid oxidation and glucose uptake in skeletal muscle (Backhed et al., 2007). On the other hand, the lack of microbiota also caused an increase in the expression of fasting-induced adipose factor (FIAF) in the intestine. This FIAF is an inhibitor of the lipoprotein lipase peroxisomal proliferator that may be involved in regulating the expression of genes encoding key enzymes implicated in fatty acid oxidation in muscle, in an AMPK-independent mechanism. Moreover, locomotor activity of the germ-free animals was higher than their counterparts. Although the cause of this increased locomotor activity is unknown, it may indicate a relation between the metabolic activity of the microbiota and behaviors that may contribute to the observed differences in adiposity between animals without microbiota and conventional animals (Backhed et al., 2004). Currently, the mechanisms by which exercise may cause changes in microbiota are not fully understood. Probably a compendium of factors and pathways are involved in the observed changes. In this review, we attempt to enumerate them (Figure 2).

### Bile acids

One of the factors by which exercise may cause changes in gut microbiota is the modification of the bile acids profile. Several studies have found an inverse relation between the amount of fecal bile acids and physical activity, and this relation becomes stronger as physical activity intensifies (Hagio et al., 1985; Sutherland et al., 1991; Wertheim et al., 2009). In general, bile acids have an antimicrobial effect, but not all to the same extent, so depending on the bile acids profile and their concentration they may exert selective pressure on certain bacterial groups, favoring the presence of some and reducing the presence of other bacterial groups. In fact, in rats whose diets were supplemented with cholic acid, a great change in microbiota profile was observed in both diversity and composition, resulting in an increase of the *Firmicutes* phylum (mainly *Clostridia* class) and decrease of the *Bacteroidetes* phylum (Islam et al., 2011). Furthermore, microbiota is capable of synthesizing the so-called secondary bile acids that can bind receptors in the liver and muscle. Bile acids, in addition to their function related to the absorption of lipids and cholesterol metabolism, can work as metabolic function integrators, activating hormone receptors such as farnesoid X receptor (FXR), that protects against body weight gain and liver and muscle fat deposition (Wang et al., 1999; Claudel et al., 2005; Cipriani et al., 2010). Watanabe et al. found that the addition of cholic acid to the diet of mice fed with a high-fat diet caused an increase in the expression of genes related to energy expenditure, mainly those related to cyclic-AMP-dependent thyroid hormone-activating enzyme type 2 iodothyronine deiodinase (D2) in brown adipose tissue (Watanabe et al., 2006) (Figure 2). Although brown adipose tissue is not found significantly in humans, D2 is significantly expressed in skeletal muscle, and so ultimately it could be hypothesized that bile acids may increase energy expenditure in muscle.

### Short-chain fatty acids

Another fact that supports the existence of a muscle-microbiota axis is the change that exercise produces in the fecal short-chain fatty acids (SCFAs) profile. In animal models, it has been observed that running exercise increases fecal butyrate levels, and this change is associated with changes in butyrate-producer bacteria groups (Matsumoto et al., 2008). Therefore, increased SCFAs production through microbiota profile changes could be one of the mechanisms by which physical exercise promotes health, since SCFA butyrate has the ability to inhibit histone deacetylases, and subsequently it has an impact on gene regulation, immune modulation, cancer suppression, cell differentiation, intestinal barrier regulation, oxidative stress reduction, diarrhea control, visceral sensitivity, and intestinal motility modulation (Leonel and Alvarez-Leite, 2012).

On the other hand, the SCFAs produced by the microbiota are capable of activating AMPK in the muscle (Yamashita et al., 2007, 2009) (Figure 2). AMPK controls the activity of various factors implicated in the regulation of cholesterol levels and in the metabolism of lipids and glucose in the muscle (den Besten et al., 2013; Kasubuchi et al., 2015). The activation of AMPK in the muscle by the SCFAs can occur directly by augmenting the AMP/ATP ratio and/or indirectly through the Ffar2-leptin pathway, but the extent to which AMPK activation is regulated for each pathway *in vivo* is still unknown (den Besten et al., 2013). Moreover, SCFAs through Ffar2/3 receptors in the colon increase plasma PYY (a satiety hormone) that reinforces the action of insulin on glucose disposal in muscle and adipose tissue (den Besten et al., 2013).

### Toll-like receptors—lipopolysaccharide

The activation of the TLRs in the muscle by lipopolysaccharide (LPS) from the membrane of bacteria is another route by which the muscle and the microbiota may be in communication. The muscles express TLR4 and TLR5 receptors that could be activated by circulating LPS (LPS9 or flagellin respectively) and whose levels depend on gut microbiota composition (Bindels and Delzenne, 2013). The stimulation of TLRs by LPS from the membrane of certain bacterial types triggers the production of inflammatory cytokines in the muscle through the activation of the nuclear factor kappa-light-chain-enhancer of activated B cells (NF-kB) (McFarlin et al., 2004; Stewart et al., 2005). In fact, the injection of LPS in mice caused muscle atrophy through TLR4 receptors (Doyle et al., 2011). Acute and chronic physical exercise in rats on a high-fat diet induced an important suppression in the TLR4 signaling pathway in the liver, muscle, and adipose tissue, reducing LPS serum levels and improved insulin signaling and sensitivity (Oliveira et al., 2011). Moreover, exercise prevented lung injury and associated oxidative stress provoked by instillation of LPS (Reis Goncalves et al., 2012; da Cunha et al., 2013, 2014) and LPS-induced depressive-like behavior in rats (Martin et al., 2014).

### Ig-A-mediated mucosal immunity

An increase of immunoglobulin A (IgA) production and a reduced number of B and CD4 + T cells have been observed in the gut of animals that performed long-term moderate exercise compared to sedentary mice. Gene expression of IL-6, IL-4, IL-10, and TGF-β cytokines (which are involved in IgA regulation) and that of TNF-α and IL-12 was overexpressed in the duodenum of exercised mice, whereas IL-2 gene expression was downregulated (Viloria et al., 2011). The increased levels of intestinal IgA caused by exercise may augment the resistance of exercised mice to intestinal pathogen infections, as well as the resistance to colonization by commensal microbiota, ultimately influencing the composition of the microbiota (Viloria et al., 2011; Macpherson et al., 2015).

### Myokines

During physical activity, myokines (cytokines and other peptides) are released from muscle fibers, exerting a paracrine and endocrine effect. Muscle cells are able to produce IL-6 by themselves, increasing up to 100-fold the circulating levels of this cytokine during exercise (Fischer, 2006). Circulating IL-6 seems to have a dual effect: an effect related to metabolism, in which IL-6 acts by increasing fat oxidation and glucose uptake via AMPK phosphorylation (van Hall et al., 2003; Pedersen and Febbraio, 2012), but also an anti-inflammatory effect, as IL-6 produced during exercise triggers the secretion of IL-10, IL-1ra, and TNF-R anti-inflammatory cytokines, protecting against chronic diseases associated with low-grade inflammation (Petersen and Pedersen, 2005). It is well known that microbiota is altered during inflammation-related diseases such as inflammatory bowel diseases, cardiovascular diseases, and diabetes (Sekirov et al., 2010); however, whether IL-6 or other myokines released from the muscle could have an impact on microbiota is a totally unexplored topic.

### Weight loss

Another factor by which exercise could cause changes in gut microbiota composition is the weight loss that sometimes is associated with exercise. Diversity and composition of microbiota from obese individuals differ from microbiota of non-obese individuals (Turnbaugh et al., 2008; Xu et al., 2012; Teixeira et al., 2013; Remely et al., 2015). However, the nature of these changes and how they are produced is unknown; if weight loss involves changes in gut microbiota or if a change in gut microbiota composition contributes to weight loss is a question that requires further research.

### Gut transit time

Moderate exercise reduces intestinal transit time (Oettle, 1991), which could influence the microbiota composition. In fact, a decrease in *Bacteroidetes* phylum and *Prevotella* genus in the microbiota of constipated obese children was observed in comparison with the microbiota of obese children with normal intestinal transit time (Zhu et al., 2014). The stool consistency (a parameter related to intestinal transit time) is strongly related to microbial diversity; stool firmness is related to *Methanobrevibacter, Oxalobacter, Butyricimonas*, and *Akkermansia* populations, whereas the *Bacteroides* genus is more abundant in loose stool (Vandeputte et al., 2015). The presence of certain genera of bacteria or others depending on the gut transit time could be explained by the adaptation of determined bacterial genera to growth in slow-transit time conditions, with reduced ecosystem water activity, resistance to water stress conditions, or higher fluctuations in nutrient availability, or, on the contrary, by the adaptation to grow in fast-transit time conditions with the ability to attach to colonic tissue or to have a high growth rate (Vandeputte et al., 2015). Besides modifying the intestinal transit, strenuous and prolonged exercise (i.e., long-distance running and triathlons) provokes diarrhea and gastrointestinal bleeding (Martin, 2011). These changes in gut permeability produce a phenomenon of ischemia and reperfusion that can affect gut microbiota. In murine models of ischemia reperfusion, a dynamic change in gut microbiota occurs; microbiota diversity decreases in early injury, whereas in reperfusion there is an increase of *Escherichia coli* and *Prevotella oralis* and a decrease of several species of the *Lactobacillus* genus (Wang et al., 2012, 2013). There are no human studies related to this issue, but it would be interesting to conduct some, given the importance of gut microbiota in health (Bermon et al., 2015).

### Stress and hypothalamic–pituitary–adrenal axis

The term microbial endocrinology was first coined by Lyte in 1993 as a “conceptual framework to understand interactions between the microbiota and the host” (Lyte, 1993; Bailey, 2014). Commensal bacteria are able to segregate hormones and neurotransmitters (epinephrine, acetylcholine, histamine, serotonin, gamma aminobutyric acid) which can induce changes at the brain level, and in turn, bacteria have receptors for these hormones, thus they can communicate with the host brain (Bailey, 2014). The activation of the HPA axis produces changes in certain populations of bacteria (Pullinger et al., 2010b), and bacteria can produce hormones that modify the behavior of the host (Bravo et al., 2011). This communication axis allows certain hormone-releasing stimuli, such as stress, to modify the gut microbiota (Figure 2). Stress of physical or psychological etiology causes HPA axis activation and the release of various hormones (corticotropin, cortisol, noradrenaline, adrenalin, dopamine) (Axelrod and Reisine, 1984), and gut microbiota dysbiosis (Galley and Bailey, 2014). The release of corticotropin releasing factor (CRF) causes changes in gastric acid secretion, gastrointestinal motility, and mucus production (Tache and Perdue, 2004; Bhatia and Tandon, 2005) that likely influence gut microbiota. In animal models, the stress caused by maternal separation or food deprivation increases serotonin and noradrenaline levels, provoking a shift in microbiota profile with a decrease of lactobacilli which increase the host susceptibility to opportunistic infections (Bailey and Coe, 1999; O'Mahony et al., 2009; Reynolds et al., 2010). Similar changes were found in gut microbiota of undergraduate students in a stressful situation like the examination period (Knowles et al., 2008). Moreover, elevated plasmatic levels of norepinephrine due to stress have an impact on gut microbiota by increasing the virulence of enteric pathogens such as *Salmonella enterica* serovar *typhimurium* and *E. coli* (Freestone et al., 2007; Pullinger et al., 2010a). In physical exercise, physical stress and homeostasis disruption occurs when the body exceeds 60% of the maximum volume of oxygen (VO2max) or the duration of exercise exceeds 90 min, even if the intensity does not exceed 40% VO2max (Luger et al., 1987), producing an activation of the HPA axis and hormone release which is more significant as the exercise intensity increases (Duclos et al., 1997). In addition to physical stress, athletes in pre-competition periods suffer high psychological stress (Noblet and Gifford, 2002) that also triggers HPA axis activation. All of the above indicates that the release of hormones that occurs during exercise could modify the microbiota profile of subjects who practice physical exercise at certain intensities or durations. However, studies are needed to confirm this hypothesis, as there are no studies in the literature addressing the effect of physical and psychological stress linked to exercise in gut microbiota.

## Perspective

There is scientific evidence that exercise has a health-promoting effect. However, if this beneficial effect may be associated in part to gut microbiota modulation, it has been barely explored. Preclinical studies indicate that physical exercise promotes microbial diversity (which is associated with a healthier state) and increases health-beneficial gut bacteria populations. Moreover, the only study carried out on humans appears to confirm murine model results. However, it is not clear which populations are modified and how, as has been shown by contradictory findings. Some of the reasons for these discrepancies could be the various forms of exercise used in these studies (acute exercise, chronic exercise, cardio and resistance training, voluntarily), the different doses of exercise applied, and the exercise duration. The observed changes in gut microbiota are triggered by underlying mechanisms which are quite unknown nowadays and that we have tried to compile. The importance of each of these factors and to what extent they could modify the gut microbiota still require further investigation. Given the close relation between the microbiota and the immune system and its involvement in several diseases, it is essential to know which factors modify gut microbiota, and exercise seems to be one of them. Manipulation of gut microbiota by modifying diet or exercise habits could be a powerful tool in the future to prevent or treat several diseases.

## Author contributions

BC, MP, ML have participated in the concept, design and critical review of the manuscript. RS, JP, and JT have draft the work. All named authors had given their approval for publication.

### Conflict of interest statement

The authors declare that the research was conducted in the absence of any commercial or financial relationships that could be construed as a potential conflict of interest.
